# Blocking Msr1 by berberine alkaloids inhibits caspase-11-dependent coagulation in bacterial sepsis

**DOI:** 10.1038/s41392-021-00483-w

**Published:** 2021-02-28

**Authors:** Chuang Yuan, Ming Wu, Qicai Xiao, Wang Zhao, Hongli Li, Yanjun Zhong, Minyi Zhao, Cheng Li, Yang Li, Xinyu Yang

**Affiliations:** 1 Department of Hematology, Xiangya Hospital, Central South University, Changsha, PR China; 2 Department of Critical Care Medicine, The Second People’s Hospital of Shenzhen & First Affiliated Hospital of Shenzhen University, Shenzhen, PR China; 3 Department of Biotherapy, State Key Laboratory of Biotherapy and Cancer Center, West China Hospital, Sichuan University and Collaborative Innovation Center, Chengdu, P R China

**Keywords:** Infectious diseases, Molecular medicine

**Dear Editor**,

Over-activation of the coagulation system in bacterial sepsis leads to disseminated intravascular coagulation (DIC), a life-threatening pathophysiological syndrome.^1^ We previously verified that deficiency of the receptor of intracellular lipopolysaccharide, caspase-11, or its upstream, type I interferons (IFNs), significantly reduces endotoxin-mediated pore-forming and phosphatidylserine exposure, which dampens the activity of TF and subsequently the coagulation cascades.^2,3^ Thus, inhibition of caspase-11 pathway may be a novel strategy for treating the endotoxin-mediated coagulation syndrome.

Here, we introduced a screening for a natural product library to identify the inhibitors of the caspase-11 pathway, in which pathophysiological outer membrane vesicles (OMVs), Gram-negative bacteria-produced vehicles, were used for delivering LPS into the cytosol to stimulate caspase-11-dependent macrophages activation.^4^ Notably, berberine alkaloids, such as berberine, palmatine, jatrorrhizine and coptisine, had highly inhibitory effects on OMV-dampened cell viability. In addition, berberine alkaloids dramatically inhibited OMV-induced cytotoxicity and augment of IL-1β in mouse macrophages or human THP-1 cells in a dose-dependent manner (Fig. 1a, b and Supplementary Fig. 1c–e). Using *Casp-11*-deficient cells as negative controls, berberine alkaloids remarkably reduced the cleaved GSDMD in OMV-treated macrophages, indicating the inhibition of the caspase-11 signalling pathway (Fig. 1c). Due to the derivation of OMV, the effect of berberine alkaloids was further determined in *E. coli*-stimulated cells. Similarly, berberine alkaloids suppressed pyroptosis in *E. coli*-treated macrophages and THP-1 cells (Supplementary Fig. S1f, S1g and S1j), and the inhibitory effects were dose-dependent (Supplementary Fig. S1h and S1i). Moreover, the derivatives, including 8-oxyberberine, 1-methoxyberberine, 13-methylberberine and 13-methylpalmatine, also attenuated OMV-mediated pyroptosis (Supplementary Fig. S1k and S1l) and GSDMD cleavage (Supplementary Fig. S1m). Together, these results indicate that berberine alkaloids and the derivatives effectively inhibit caspase-11 pathway.

**Fig. 1:**
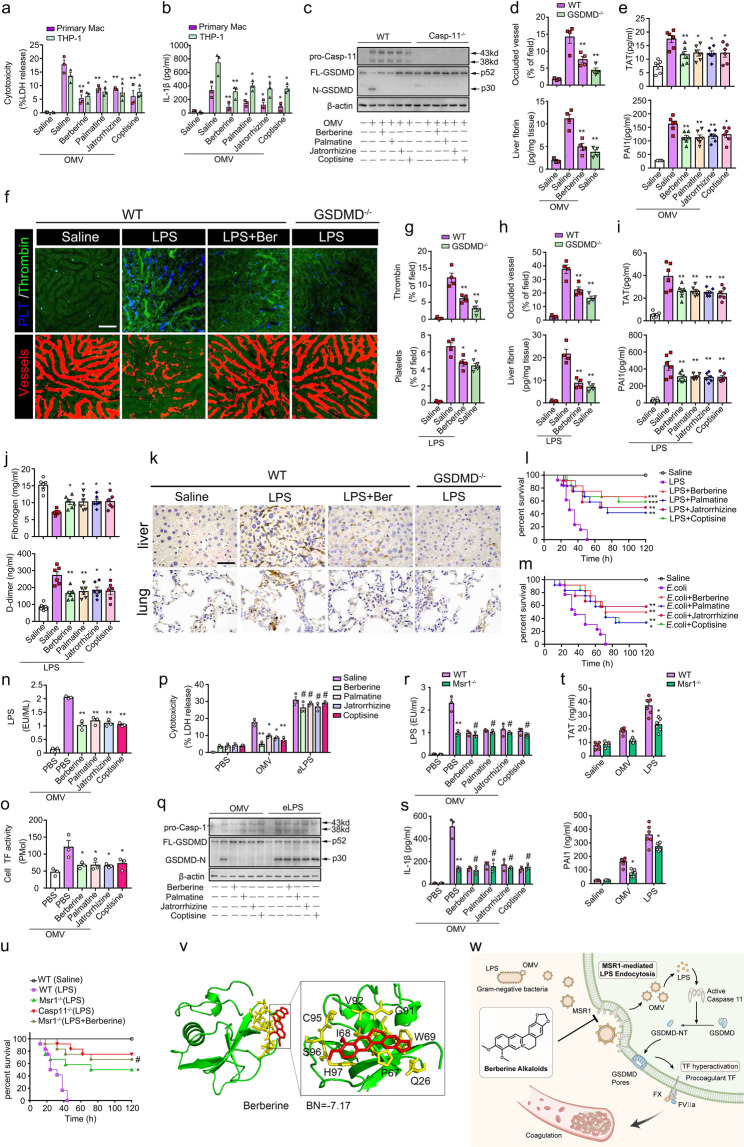
Berberine alkaloids target Msr1 to inhibit endocytosis of LPS and attenuate caspase-11-mediated coagulation activation in bacterial sepsis. **a**, **b** Cytotoxicity (%LDH release, (**a**)) and IL-1β (**b**) in supernatants of macrophages or human THP-1 cells treated with PBS or berberine alkaloids (5 μM) and challenged with OMV (10 μg/ml) (versus PBS + OMV groups). **c** Western blotting indicating caspase-11 and cleavage of GSDMD in macrophages (WT and *Casp11*^−/−^). **d**, **e** Mice were primed with LPS (0.4 mg/kg) for 7 h and subsequently administrated with berberine alkaloids (5 mg/kg) 30 min before a challenge of OMV (30 mg/kg) (versus saline-treated plus OMV-challenged WT groups). **d** Quantitative analyses of occluded microvasculature in the liver using ImageJ and fibrin deposition using ELISA. **e** Plasma levels of TAT and PAI-1. **f**–**k** Mice were primed with LPS (0.4 mg/kg) for 7 h and subsequently administrated with berberine alkaloids (5 mg/kg) 30 min before a challenge of LPS (4 or 10 mg/kg) (versus Saline-treated plus LPS-challenged WT groups). **f** Representative SD-IVM images indicating active thrombin (green), platelet aggregation (blue) and vessel occlusion (red) in the liver microvasculature (Bar = 50 μm). **g** Quantitative analyses of thrombin-loaded and platelet-aggregated microvasculature in the liver using ImageJ. **h** Quantitative analyses of occluded microvasculature in the liver using ImageJ and fibrin deposition using ELISA. **i** Plasma levels of TAT and PAI-1. **j** Plasma levels of fibrinogen and D-dimer. **k** Representative image of Immunohistochemical staining of fibrin in the liver and the lung (×400). **l**, **m** Kaplan–Meier survival plots in mice treated with berberine alkaloids (5 mg/kg) 30 min prior to the challenge of 10 mg/kg LPS (**l**) or *E.coli* (**m**) (versus Saline + challenge group). **n**, **o** Cytosolic levels of LPS (**n**) and TF activity (**o**) in macrophages treated with berberine alkaloids (2 μM) and OMV (10 μg/ml) (versus Saline + OMV group). **p**–**s** Macrophages were treated with berberine alkaloids (2 μM) and challenged by OMV (10 μg/ml) or an electrorotation of LPS (1 μg/10^6^ cells) (versus Saline + OMV or LPS electrorotation groups, respectively). **p** Cytotoxicity (%LDH release). **q** Western blotting indicating caspase-11 and cleavage of GSDMD. **r**, **s** Levels of cytosolic LPS (**r**) and IL-1β release (**s**) in WT and *Msr1*-deficient macrophages (WT groups versus *Msr1*^−/−^ groups). **t** Plasma TAT and PAI-1 in WT and *Msr1*-deficient mice challenged with OMV or LPS (WT groups versus *Msr1*^−/−^ groups). **u** Kaplan–Meier survival plots in WT, *Msr1*^−/−^ and *Casp11*^−/−^ mice challenged with LPS in the presence or absence of berberine (versus *Msr1*^−/−^ groups). **v** Binding energy of berberine alkaloids to Msr1 in molecular docking. **w** The mechanism that berberine alkaloids inhibit endotoxin-mediated coagulation activation. *p < 0.05; **p < 0.01, # no significant difference. Data are shown as mean ± SEM

Next, we validated our in-vitro findings in OMV-challenged mice. With *Gsdmd*-deficient mice as negative controls, berberine markedly dampened vessel occlusion and fibrin deposition in liver microvasculature (Fig. 1d and Supplementary Fig. S2a). Berberine alkaloids also significantly attenuated DIC markers (Fig. 1e). As the endotoxemia model is widely used in the study of caspase-11, we further determined the protective effects of berberine alkaloids in mice challenged with LPS. Similar to deficiency of GSDMD, the downstream of caspase-11, berberine significantly alleviated the endotoxin-mediated thrombin generation, platelet aggregation and vessel occlusion throughout the liver microvasculature (Fig. 1f–h). The DIC markers, such as augment of PAI-1, TAT and D-dimer, consumption of fibrinogen, or fibrin deposition, were also inhibited by berberine alkaloids or the derivatives (Fig. 1h–k and Supplementary Fig. S3). To mimic the clinical practice, the clinic-relevant Gram-negative sepsis models, including intraperitoneal *E. coli* and CLP, were used. The inhibitory effects of berberine alkaloids on coagulation syndrome were phenocopied in mice subjected to *E. coli* or CLP (Supplementary Fig. S2b–g). Excessive coagulation activation in sepsis may result in organ dysfunction or death. Berberine alkaloids dramatically attenuated multi-organ dysfunction and death rate in mice challenged with LPS, *E. coli* or CLP (Fig. 1l, m and Supplementary Fig. S4a–g). Taken together, berberine alkaloids and the derivatives, inhibiting the caspase-11 pathway, are effective medicine to attenuate coagulation activation, organ dysfunction and lethality in bacterial sepsis.

How berberine alkaloids inhibit the caspase-11 pathway? Cytosolic accessing as well as binding to LPS is required for caspase-11 activation. Berberine alkaloids significantly reduced the cytosolic translocation of LPS and the cytosolic colocalization of LPS and caspase-11 (Fig. 1n and Supplementary Fig. S5a and S5b). To assess if berberine alkaloids affect LPS/caspase-11 binding, LPS was physically translated into the cytosol using electroporation. Berberine alkaloids effectively retrieved OMV-mediated but not electroporated-LPS-induced pyroptosis (Fig. 1p, q, and Supplementary Fig. S5c and S5d). Thus, berberine alkaloids inhibit the caspase-11 pathway by suppressing the cytosolic translocation of LPS rather than the binding of LPS and caspase-11.

As we previously indicated, caspase-11 activation triggers TF activity and consequently leads to coagulation syndrome.^5^ In agreement with our previous study, berberine alkaloids diminished OMV-increased TF activity (Fig. 1o), but did not alter TF expression (Supplementary Fig. S5f and S5g). Activation of TF initiates the extrinsic coagulation cascade that leads to thrombin formation. Accordingly, the highly upgraded thrombin in the OMV-treated group was restored when berberine alkaloids were administrated (Supplementary Fig. S5e). Together, the protection of berberine alkaloids against coagulation activation attributes to their inhibition to caspase-11-dependent TF activity.

Clathrin-dependent endocytosis is implicated as a key pathway in the cytosolic translocation of LPS.^4^ In addition, LPS receptors are also associated with LPS internalization of OMV. Thus, the target of berberine was screened from the components of clathrin-dependent endocytosis or LPS-binding factors using molecular docking and validated by silencing selected genes. Knockdown of AP2,^4^ Integrin α5, Cd14 or Msr1 alone inhibited cytosolic LPS, the release of IL-1β and augment of TF activity or thrombin in OMV-challenged macrophages (Supplementary Fig. S6a–h). Berberine exerted an additive inhibition in the macrophages with down-regulated AP2, Integrin α5 and CD14 but not Msr1 (Supplementary Fig. S6a–h). In addition, Msr1 knockout dramatically inhibited the cytosolic translocation of LPS and augment of IL-1β (Fig.1r and s). Berberine alkaloids did not further improve the inhibition in *Msr1*-deficient cells (Fig. 1r and s). Similar to the administration of berberine alkaloids, *Msr1* deficiency suppressed caspase-11 signalling and cleavage of GSDMD (Supplementary Fig. S6i), and consequently reduced pro-coagulant property in thrombin formation when did not affect the expression of TF (Supplementary Fig. S6j–l). In line with the in vitro experiments, coagulation activation remarkably dropped in *Msr1*-deficient mice after a challenge of OMV or LPS (Fig. 1t). Administration of berberine did not additively affect the improvement in the lethality of mice (Fig. 1u). Moreover, berberine alkaloids showed high binding energy with Msr1 (Fig. 1v and Supplementary Table S2). Taken together, berberine alkaloids, at least in part, target Msr1 to inhibit endocytosis of LPS and caspase-11-mediated coagulation activation (Fig. 1w).

Msr1 is a scavenger receptor (SR) facilitating endocytosis of modified low-density lipoprotein and pathogens. We revealed a novel role of Msr1 that mediates endocytosis of LPS and consequently activates caspase-11. SRs are a family that functions in the engulf of pathogens by immune cells. Knockout of SRs is previously reported to be protective and would be a target for treating sepsis. Thus, other SRs may also be the mediator of LPS internalization, which remains to be investigated in further study. Anti-coagulant drugs are applied in certain selected patients diagnosed with DIC. Early intervention using anti-coagulant drugs may interrupt the hemostasis of physiological haemostasis and immunothrombosis in the defense of pathogens, and not be recommended in clinical practice. Blocking the key molecular that initiates the blood coagulation cascade may be an optional and additive strategy for preventing coagulation syndrome. In line with our previous study, we found that berberine and the structural analogs, inhibiting caspase-11 pathway by blocking Msr1, significantly attenuate coagulation activation in bacterial sepsis. Berberine is safely and traditionally used for treating diarrhoea and enteritis for centuries. In addition, berberine alkaloids possess anti-microbial and anti-inflammatory activity. Given that severe infection and over-inflammation are common in sepsis, berberine would be an optimal molecular skeleton in the development of a drug to treat coagulation syndrome in septic patients.

In conclusion, Msr1 is a novel mediator of endocytosis of LPS that activates the caspase-11 pathway and berberine alkaloids serve as the inhibitors. Msr1 would be a new target and berberine alkaloids could be candidate drugs in the prevention and treatment of coagulation syndrome in sepsis.

## Supplementary information

Supplemental Materials

## Data Availability

All data and materials are included in the article. Further information can be obtained from the corresponding author (Xinyu Yang, yangxinyu@csu.edu.cn) upon request.
