# Supplementary material for: Blocking Msr1 by berberine alkaloids inhibits caspase-11-dependent coagulation in bacterial sepsis
Source: Signal Transduct Target Ther. 2021 Feb 28;6:92. doi: 10.1038/s41392-021-00483-w (PMC7914254; doi:10.1038/s41392-021-00483-w)
Supplement: Supplementary file 1 — Supplemental Materials [file 41392_2021_483_MOESM1_ESM.docx]

Supplementary Materials for

**Blocking Msr1 by Berberine Alkaloids Inhibits Caspase-11-dependent Coagulation in Bacterial Sepsis**

Chuang Yuan ^1^, Ming Wu ^2^, Qicai Xiao ^3, 4^, Wang Zhao ^1^, Hongli Li ^1^, Yanjun Zhong ^5^, Minyi Zhao ^6^, Cheng Li ^7^, Yang Li ^8^, Xinyu Yang ^1, *^

*Correspondence to Dr. Xinyu Yang: [yangxinyu@csu.edu.cn](mailto:yangxinyu@csu.edu.cn)

This PDF file includes:

Materials and Methods

Reference for ‘Materials and Methods’

Tables. S1 and S2

Figures. S1 to S6

**Methods and materials**

**Reagents**

Selleck provided the natural products library (L1400). The derivatives, including 8-oxyberberine (CB2160647), 1-methoxyberberine (CB51239484), 13-methylberberine (CB82266822) and 13-methylpalmatine (CB61178432), were obtained from Chengdu Purechem-Standard Co., Ltd. CCK8 Assay kit and LDH Assay kit were used for assessing cell viability and cytotoxicity, respectively. Commercial ELISA kits were used for determining the concentration of target proteins, including mouse IL-1α (Cat: 88-5019, Invitrogen), mouse IL-1β (Cat: 88-7013, Invitrogen), human IL-1α (DY200, R&D), human IL-1β (Cat: 88-7261, Invitrogen), mouse D-dimer (Cat: CEA506Mu, Cloud-Clone), mouse fibrinogen (Cat: ab108844, Abcam), mouse PAI-1 (Cat: ab197752, Abcam), mouse TAT (Cat: ab137994, Abcam), mouse fibrin (MBS706338, MybioSource) and TF (DCF300, R&D Systems; ab214091, Abcam). Anti-mouse fibrin antibodies (59D8) were kindly provided by Professor Nigel Mackman. TF activity was detected using the Assay Sense Human Tissue Factor Chromogenic Activity Kit (CT1002b). Thrombin formation was assessed using SensoLyte internally quenched 5-FAM/QXL-520 FRET thrombin substrate from Anaspec (Cat.AS-72129). Western Blot were conducted using antibodies against caspase-11 (Cat: C1354, Sigma), mouse GSDMD (Cat: ab209845, Abcam) and mouse β-actin (clone 8H10D10, Cat: 3700S, Cell Signaling Technologies). LPS derived from E.coli 0111：B4 was purchased from Sigma (L2630). OMV was isolated as previously reported ^1^.

**Mice and Models of Endotoxemia and Bacterial Sepsis**

*Casp11*^-/-^ (Jackson Laboratory, 024698), *Gsdmd*^-/-^ (Jiahuai Han Lab), *Msr1*^-/-^ (Jackson Laboratory, 006096), *HCV* mice (express human tissue factor, Nigel Mackman Lab) and WT littermate male mice in C57BL/6J background with body weight of 25-30 g were used in the present study. Mice were primed using 0.4 mg/kg LPS for 7 hours and subsequently challenged by 30 mg/kg OMV or 10 mg/kg LPS to generate the DIC-like model ^2-4^. After priming, berberine alkaloids and the derivatives were intraperitoneally introduced 30 min prior to the challenge of OMV or LPS. Mice were sacrificed and the tissues and the plasma were obtained at 8 hours after the 30 mg/kg OMV or 10 mg/kg LPS challenge. To determine the survival rate, mice were treated with saline or the chemicals 30 min before a challenge of the 10 mg/kg LPS (with priming), 10^9^ CFU E.coli or cecal ligation and puncture (CLP). In the intravital microscopy, 4 mg/kg LPS after the 0.4 mg/kg LPS priming were intraperitoneally injected to mice for 6 hours, which allows the majority of blood vessels to be not completely occluded for better visualization of the liver microcirculation ^4,5^. All mice were maintained under standard condition (room temperature 22-25°C with a 12-h light-dark cycle) and were allowed to free access to water and standard chow. All animal experiments of this study were approved by the Research Ethics Committee of Xiangya Hospital and the Institutional Animal Care and Use Committees of Central South University.

**Cecal Ligation and Puncture (CLP)**

The cercum was exposed after a 1.5cm longitudinal midline incision in mice anesthetized by 2% isoflurance (Piramal Critical Care) with oxygen. A severe polymicrobial sepsis model was conducted by ligating 75% of the cecum and extruding a small amount of feces from a through-and-through puncture made by 18-gauge needles. The cecum was relocated and the abdomen was closed. Pre-warmed normal saline (37 °C; 5 ml per 100 g body weight) was subcutaneously injected to allow mice to recover from anaesthetization. Sham-operated animals were subjected to same procedure except the ligation and puncture.

**Intravital microscopy and image analysis**

After anesthetized using xylazine hydrochloride (10 mg/kg) and ketamine hydrochloride (200 mg/kg), mice were injected with the thrombin substrate or antibodies via the right jugular vein (2μl thrombin substrate, 5 μl AF647/anti-CD49b antibody or 0.05 μg AF647-conjugated anti-mouse albumin antibody). The left liver was subsequently exposed and externalized on a thin glass coverslip that loaded on an inverted microscope heat-controlled stage of spinning disk confocal intravital microscopy. Fluorescent images were obtained and analyzed as previously described ^4,5^.

**Cell culture**

Mouse peritoneal macrophages were isolated from caspase-11 KO, HCV (human TF positive) or WT C57BL/6J male mice as previously shown ^4,5^, and loaded on 96- or 12-well plates or 6-well slides overnight. After the treatment of 0.5, 1, 2 or 5 μM chemicals, macrophages were challenged with OMV (10 μg/ml) or *E.coli* (MOI 10, 2 hours), and cultured for 16 hours until harvested. Silencing AP2, Integrin α5, CD14 and Msr1 in macrophage using siRNA was conducted according the instruction of manufacturer (Sango Biotech). Stimulated with 20 ng/ml of PMA (Sigma), human THP-1 cells were plated on 96- or 12-well plates overnight. Chemicals at a dose of 0.5, 1, or 2 μM were given prior to the challenge of OMV (10 μg/ml) or *E.coli* (MOI 10, 2 hours).

**Proximity-ligation assay**

The interaction of LPS and caspase-11 was determined using Proximity Ligation Assay kit (PLA, Sigma), a technique to visualize the molecular-molecular colocalization. Briefly, macrophages loaded on six-well slides were stimulated with OMV and/or treated with berberine alkaloids or the derivatives for 2 hours. After fixed with 4% formaldehyde and permeabilized by Triton X-100, cells were incubated at 4 ^o^C overnight with primary antibodies to LPS (mouse monoclonal 2D7/1, Abcam, ab35654) or caspase11 (rat monoclonal 17D9, Sigma-Aldrich, C1354). In situ PLA was conducted based on manufacturer’s instructions. Nikon confocal laser scanning microscope was used to take images that were quantified using Image-J software.

**Tissue histology**

After perfused with phosphate-buffered saline (PBS) containing heparin (20 IU/ml) and fixed using 10% formalin, the liver (left lobe) were embedded in paraffin. The sections with a thickness of 4 μm were obtained for hematoxylin and eosin staining and immunohistochemistry (IHC). In the IHC to determine fibrin, the sections were incubated with anti-mouse fibrin antibody at a dilution 1:500 at 4 ^o^C overnight. Four times of washes using PBST (PBS containing 0.1% tween-20) were conducted between steps. The sections were subsequently incubated with anti-mouse HRP-conjugated secondary antibody and fibrin-positive signals were visualized using 3, 3′-diaminobenzidine tetrahydrochloride reaction.

**Western blot**

The liver was perfused with cold PBS prior to harvest for western blot. Lysis of tissues or cells in RIPA was conducted using ultrasonication at 4 ^o^C. Supernatants after a centrifuge at 10, 000 g for 10 min were obtained for further experiments. Proteins were transferred to PVDF membranes after SDS-polyacrylamide gel electrophoresis. The membranes were blocked using 5% fat-free milk and incubated with primary antibodies at a dilution of 1:1000 (except 1:5000 for β-actin) at 4 ^o^C overnight. Afterward, the membranes were incubated with HRP-conjugated secondary antibody (1:5000) at room temperature for 2 hours. Four times of washes using TBST were conducted between steps. The blots were visualized by Western Bright ECL-Spray (advansta, catalog number: K-12049-D50) using Bio-Rad system。

**qRT-PCR**

According to the manufacturer's instructions, total RNA was purified from cells using Trizol (Life Technologies, Gaithersburg, MD). mRNA was reversely transcribed for cDNA using Reverse Transcription Kit (Cat #k1622, Thermo Fisher Scientific, USA). The cDNA of target gene was amplified using TaqMan Universal PCR master mix (Applied Biosystems, Foster City, CA, USA) and the primers as follow: TF, 5’-AACCCACCAACTATACCTACACT-3’ and 5’-GTCTGTGAGGTCGCACTCG-3’; Msr1, 5’-TGGAGGAGAGAATCGAAAGCA-3’ and 5’-CTGGACTGACGAAATCAAGGAA-3’; Integrin α5, 5’-CCGTGGACTTCTTCGAGCC-3’ and 5’-CTGTTGAATCAAACTCAATGGGC-3’; CD14, 5’-CTCTGTCCTTAAAGCGGCTTAC-3’ and 5’-GTTGCGGAGGTTCAAGATGTT-3’. After normalized by the amplification of glyceraldehyde-3-phosphate dehydrogenase (GAPDH), the expression levels of genes were shown as the fold changes relative to the control groups.

**TF activity and** **thrombin assay**

THP-1 cells stimulated with PMA (20 ng/ml) or human TF positive macrophages were plated in 96-well plates. After the incubation of chemicals and OMV, the cells were washed 3 times using PBS and incubated with a 70 μl assay mixture containing factor VII (10 μl) and factor X (10 μl) at 37 ^o^C for 30 minutes. Afterward, 20 μl of factor Xa substrate was added to the wells. The absorbance was assessed at 405 nm every 5 minutes for 25 minutes. TF activity was determined based on the standard curve. Thrombin generation assay was used as shown in our previous study ^4^. Briefly, 10 μl cell supernatant were mixed with diluted platelet poor plasma as previously indicated. The mixture was subjected to 50 μl thrombin substrate and the fluorescence intensity was determined using TECAN multifunctioinal fluorescent enzyme-labeled instrument.

**Molecular docking**

The crystal structure of clathrin-dependent endocytosis components or LPS-related proteins were retrieved from http://www.rcsb.org/ (Table S1). Original ligands, if any, were removed from the proteins by PyMol software and the remaining molecules were saved as PDB files. The structural formulas of berberine and other candidate ligands were constructed using Chim3D (2010) software. Molecular docking between ligands and proteins was analyzed by AutoDock 4.0 software and AutoDock Tools 1.5.6 software. Independent docking calculation for ligands with proteins were conducted with 250,0000 evaluations using Lamarckian Genetic Algorithm.

**Statistical analyses**

Data are expressed as mean ± standard error of the mean for multiple experiments. GraphPad Prism 7.0 was used for the statistical analyses. Comparisons between two groups were conducted using 2-tailed student *t* test. One-way ANOVA with Benferroni’s post hoc test was used when comparisons among more than two groups were needed. The log-rank test was utilized to analyze the survival rate in mice from various groups. P values less than 0.05 were considered as statistical significance.

**References**

1 Vanaja, S. K. *et al.* Bacterial Outer Membrane Vesicles Mediate Cytosolic Localization of LPS and Caspase-11 Activation. *Cell*. **165**, 1106-1119, (2016).

2 Hagar, J. A. *et al.* Cytoplasmic LPS activates caspase-11: implications in TLR4-independent endotoxic shock. *Science*. **341**, 1250-1253, (2013).

3 Kayagaki, N. *et al.* Noncanonical inflammasome activation by intracellular LPS independent of TLR4. *Science*. **341**, 1246-1249, (2013).

4 Yang, X. *et al.* Bacterial Endotoxin Activates the Coagulation Cascade through Gasdermin D-Dependent Phosphatidylserine Exposure. *Immunity*. **51**, 983-996 e986, (2019).

5 Yang, X. *et al.* The role of type 1 interferons in coagulation induced by gram-negative bacteria. *Blood*. **135**, 1087-1100, (2020).

6 Cheng, C. *et al.* The scavenger receptor SCARA1 (CD204) recognizes dead cells through spectrin. *The Journal of biological chemistry*. **294**, 18881-18897, (2019).

7 Feinberg, H., Taylor, M. E. & Weis, W. I. Scavenger receptor C-type lectin binds to the leukocyte cell surface glycan Lewis(x) by a novel mechanism. *The Journal of biological chemistry*. **282**, 17250-17258, (2007).

8 Ojala, J. R. *et al.* Crystal structure of the cysteine-rich domain of scavenger receptor MARCO reveals the presence of a basic and an acidic cluster that both contribute to ligand recognition. *The Journal of biological chemistry*. **282**, 16654-16666, (2007).

9 Hsieh, F. L. *et al.* The structural basis for CD36 binding by the malaria parasite. *Nature communications*. **7**, 12837, (2016).

10 Dang, M. *et al.* Molecular mechanism of SCARB2-mediated attachment and uncoating of EV71. *Protein & cell*. **5**, 692-703, (2014).

11 Ma, H. *et al.* The Crystal Structure of the Fifth Scavenger Receptor Cysteine-Rich Domain of Porcine CD163 Reveals an Important Residue Involved in Porcine Reproductive and Respiratory Syndrome Virus Infection. *Journal of virology*. **91**, (2017).

12 Pearson, M. A., Reczek, D., Bretscher, A. & Karplus, P. A. Structure of the ERM protein moesin reveals the FERM domain fold masked by an extended actin binding tail domain. *Cell*. **101**, 259-270, (2000).

13 Wu, B. *et al.* Structures of the CXCR4 chemokine GPCR with small-molecule and cyclic peptide antagonists. *Science (New York, N.Y.)*. **330**, 1066-1071, (2010).

14 Kelker, M. S. *et al.* Crystal structure of human triggering receptor expressed on myeloid cells 1 (TREM-1) at 1.47 A. *Journal of molecular biology*. **342**, 1237-1248, (2004).

15 Ohto, U., Miyake, K. & Shimizu, T. Crystal structures of mouse and human RP105/MD-1 complexes reveal unique dimer organization of the toll-like receptor family. *Journal of molecular biology*. **413**, 815-825, (2011).

16 Kelley, S. L., Lukk, T., Nair, S. K. & Tapping, R. I. The crystal structure of human soluble CD14 reveals a bent solenoid with a hydrophobic amino-terminal pocket. *Journal of immunology (Baltimore, Md. : 1950)*. **190**, 1304-1311, (2013).

17 Somers, W. S., Tang, J., Shaw, G. D. & Camphausen, R. T. Insights into the molecular basis of leukocyte tethering and rolling revealed by structures of P- and E-selectin bound to SLe(X) and PSGL-1. *Cell*. **103**, 467-479, (2000).

18 Wedepohl, S. *et al.* Reducing Macro- and Microheterogeneity of N-Glycans Enables the Crystal Structure of the Lectin and EGF-Like Domains of Human L-Selectin To Be Solved at 1.9 Å Resolution. *Chembiochem : a European journal of chemical biology*. **18**, 1338-1345, (2017).

19 Preston, R. C. *et al.* E-selectin ligand complexes adopt an extended high-affinity conformation. *Journal of molecular cell biology*. **8**, 62-72, (2016).

20 Kim, H. M. *et al.* Crystal structure of the TLR4-MD-2 complex with bound endotoxin antagonist Eritoran. *Cell*. **130**, 906-917, (2007).

21 Ohto, U., Fukase, K., Miyake, K. & Satow, Y. Crystal structures of human MD-2 and its complex with antiendotoxic lipid IVa. *Science (New York, N.Y.)*. **316**, 1632-1634, (2007).

22 Sorrell, F. J. *et al.* Family-wide Structural Analysis of Human Numb-Associated Protein Kinases. *Structure (London, England : 1993)*. **24**, 401-411, (2016).

23 Ford, M. G., Jenni, S. & Nunnari, J. The crystal structure of dynamin. *Nature*. **477**, 561-566, (2011).

24 Zeng, X. *et al.* Structural insights into lethal contractural syndrome type 3 (LCCS3) caused by a missense mutation of PIP5Kγ. *The Biochemical journal*. **475**, 2257-2269, (2018).

25 Pylypenko, O. *et al.* The PX-BAR membrane-remodeling unit of sorting nexin 9. *The EMBO journal*. **26**, 4788-4800, (2007).

26 Ohwada, J. *et al.* Discovery and biological activity of a novel class I PI3K inhibitor, CH5132799. *Bioorganic & medicinal chemistry letters*. **21**, 1767-1772, (2011).

27 Trésaugues, L. *et al.* Structural basis for phosphoinositide substrate recognition, catalysis, and membrane interactions in human inositol polyphosphate 5-phosphatases. *Structure (London, England : 1993)*. **22**, 744-755, (2014).

28 Partlow, E. A. *et al.* A structural mechanism for phosphorylation-dependent inactivation of the AP2 complex. *eLife*. **8**, (2019).

29 Day, J., Passecker, A., Beck, H. P. & Vakonakis, I. The Plasmodium falciparum Hsp70-x chaperone assists the heat stress response of the malaria parasite. *FASEB journal : official publication of the Federation of American Societies for Experimental Biology*. **33**, 14611-14624, (2019).

30 Chaikuad, A. *et al.* Structure of cyclin G-associated kinase (GAK) trapped in different conformations using nanobodies. *The Biochemical journal*. **459**, 59-69, (2014).

31 Ma, L. *et al.* Transient Fcho1/2⋅Eps15/R⋅AP-2 Nanoclusters Prime the AP-2 Clathrin Adaptor for Cargo Binding. *Developmental cell*. **37**, 428-443, (2016).

32 Jia, Y. *et al.* Overcoming EGFR(T790M) and EGFR(C797S) resistance with mutant-selective allosteric inhibitors. *Nature*. **534**, 129-132, (2016).

33 Jackson, L. P. *et al.* A large-scale conformational change couples membrane recruitment to cargo binding in the AP2 clathrin adaptor complex. *Cell*. **141**, 1220-1229, (2010).

34 Muenzner, J., Traub, L. M., Kelly, B. T. & Graham, S. C. Cellular and viral peptides bind multiple sites on the N-terminal domain of clathrin. *Traffic (Copenhagen, Denmark)*. **18**, 44-57, (2017).

35 Ahmad, K. F. & Lim, W. A. The minimal autoinhibited unit of the guanine nucleotide exchange factor intersectin. *PloS one*. **5**, e11291, (2010).

36 Xia, W. & Springer, T. A. Metal ion and ligand binding of integrin α5β1. *Proceedings of the National Academy of Sciences of the United States of America*. **111**, 17863-17868, (2014).

37 Sen, M., Yuki, K. & Springer, T. A. An internal ligand-bound, metastable state of a leukocyte integrin, αXβ2. *The Journal of cell biology*. **203**, 629-642, (2013).

38 Xiong, J. P. *et al.* Crystal structure of the extracellular segment of integrin alpha Vbeta3 in complex with an Arg-Gly-Asp ligand. *Science (New York, N.Y.)*. **296**, 151-155, (2002).

39 Zhu, J. *et al.* Structure of a complete integrin ectodomain in a physiologic resting state and activation and deactivation by applied forces. *Molecular cell*. **32**, 849-861, (2008).

**Table S1. Molecular docking between berberine and proteins**

| Protein | PDB ID | FR | NP | | | CGB | | | BN | Ref |
| --- | --- | --- | --- | --- | --- | --- | --- | --- | --- | --- |
|  |  |  | X | Y | Z | X | Y | Z |  |  |
| SR-A1  (Msr1) | 6J02 | ILE68 | 60 | 40 | 40 | 16.009 | -18.603 | -3.570 | -7.17 | ^6^ |
| SR-A4 | 2OX9 | PHE720 | 60 | 60 | 60 | 77.764 | 75.829 | 49.905 | -4.63 | ^7^ |
| SR-A6 | 2OY3 | GLU511 | 60 | 60 | 60 | 5.534 | -2.053 | 17.433 | -5.97 | ^8^ |
| SR-B2 | 5LGD | SER160 | 60 | 60 | 60 | -40.505 | -34.565 | 52.944 | -4.43 | ^9^ |
| SR-B3 | 4TW2 | LEU187 | 60 | 60 | 60 | 14.856 | 40.351 | -0.512 | -4.85 | ^10^ |
| SR-I1 | 5JFB | SER487 | 60 | 60 | 60 | 15.793 | 8.693 | -5.516 | -4.58 | ^11^ |
| Moesin | 1EF1 | PHE267 | 60 | 60 | 60 | 12.053 | 123.958 | 59.384 | -3.35 | ^12^ |
| CXCR4 | 3OE8 | ASP187 | 90 | 96 | 70 | -49.203 | 42.515 | 20.171 | -3.78 | ^13^ |
| TREM-1 | 1SMO | TYR90 | 60 | 60 | 60 | 17.683 | 42.527 | 3.784 | -3.60 | ^14^ |
| MD-1 | 3B2D | TYR122 | 60 | 60 | 60 | 32.480 | -28.756 | -12.370 | -6.21 | ^15^ |
| RP105 | 3B2D | ASP257 | 78 | 80 | 66 | 38.045 | -10.952 | -10.917 | -4.96 | ^15^ |
| CD14 | 4GLP | PHE69 | 94 | 60 | 90 | 47.926 | 57.119 | 0.766 | -7.15 | ^16^ |
| P-selectin | 1G1Q | TRP76 | 88 | 88 | 60 | 64.333 | -2.417 | 16.694 | -3.39 | ^17^ |
| L-selectin | 5VC1 | LYS55 | 60 | 90 | 90 | -4.319 | 36.635 | 39.877 | -5.94 | ^18^ |
| E-selectin | 4C16 | ASN105 | 88 | 82 | 92 | 38.404 | 18.281 | 4.193 | -6.02 | ^19^ |
| TLR4 | 2Z63 | HIS229 | 78 | 86 | 122 | 19.487 | -36.555 | 4.372 | -6.55 | ^20^ |
| MD-2 | 2E59 | GLU92 | 60 | 60 | 60 | -6.205 | 19.791 | 13.202 | -6.82 | ^21^ |
| AAK1 | 4WSQ | GLN133 | 60 | 60 | 60 | -4.287 | -15.223 | -76.878 | -5.77 | ^22^ |
| Dynamin-1 | 3ZVR | CYS607 | 84 | 84 | 102 | -101.249 | 31.093 | -101.455 | -4.91 | ^23^ |
| PIP5K | 6CMW | ASP378 | 60 | 60 | 60 | -20.17 | 12.453 | -17.267 | -5.70 | ^24^ |
| SNX9 | 2RAK | ARG286 | 98 | 104 | 60 | 11.695 | 35.679 | 21.044 | -6.38 | ^25^ |
| PI3K | 3APD | ASP884 | 66 | 62 | 66 | 49.364 | 18.025 | 29.674 | -7.05 | ^26^ |
| OCRL | 4CMN | LYS491 | 96 | 94 | 104 | -17.849 | 28.500 | -18.827 | -6.39 | ^27^ |
| NECAP2 | 6OWO | SER88 | 60 | 60 | 60 | -1.389 | 1.417 | -5.194 | -5.48 | ^28^ |
| HSP70 | 6S02 | THR43 | 60 | 60 | 60 | -4.472 | 3.611 | 24.056 | -6.91 | ^29^ |
| GAK | 4C58 | CYS190 | 60 | 60 | 68 | 67.651 | 61.363 | 16.506 | -7.84 | ^30^ |
| FCHO1 | 5JP2 | THR914 | 60 | 60 | 60 | -12.167 | -21.222 | -18.444 | -4.72 | ^31^ |
| EGFR | 5D41 | THR854 | 60 | 60 | 60 | -30.229 | 28.137 | 20.857 | -6.84 | ^32^ |
| AP2 | 2XA7 | LEU336 | 60 | 60 | 60 | 56.342 | -43.595 | -7.71 | -6.75 | ^33^ |
| Clathrin | 5M5R | TPR164 | 40 | 40 | 40 | -8.301 | -25.356 | 15.786 | -6.63 | ^34^ |
| Intersectin-1 | 3JV3 | ASN1176 | 60 | 60 | 60 | -9.944 | -5.417 | -10.611 | -5.25 | ^35^ |
| INTEGRIN β1 | 4WK2 | SER227 | 100 | 88 | 56 | 16.859 | 15.159 | -30.528 | -6.85 | ^36^ |
| INTEGRIN β2 | 4NEH | ASP151 | 118 | 106 | 104 | -49.375 | 38.635 | 63.056 | -5.94 | ^37^ |
| INTEGRIN β3 | 1M1X | PHE223 | 104 | 104 | 92 | 17.766 | 28.798 | 34.860 | -5.88 | ^38^ |
| INTEGRIN αⅤ | 3FCS | TYR190 | 126 | 80 | 118 | -83.844 | -65.002 | -87.105 | -8.42 | ^38^ |
| INTEGRIN αⅡ | 1M1X | TYR178 | 126 | 126 | 84 | 8.960 | 45.838 | 31.289 | -7.02 | ^39^ |

AAK1: AP2-associated protein kinase 1; BN: binding energy (kcal/mol); CGB: center grid box; EGFR: Epidermal growth factor receptor; FCHO1: FR: flexible residues; GAK: Cyclin-G-associated kinase; HSP70: Heat shock protein 70; NECAP2: Adaptin ear-binding coat-associated protein 2; NP: number of points; OCRL: PIP5K: Phosphatidylinositol-4-phosphate 5-kinase; PI3K:Phosphatidylinositol-4,5-bisphosphate 3-kinase; Ref: references; SNX9:Sorting nexin-9; TLR: Toll-like receptor.

**Table S2. Molecular docking between berberine alkaloids as well as the derivatives and Msr1**

| Ligand | Structure | Interaction residues | BN |
| --- | --- | --- | --- |
| Berberine |  | GLN26, PRO67, ILE68, TRP69, GLY91, VAL92, CYS95, SER96, HIS97 | -7.17 |
| Palmatine |  | GLN26, GLY66, PRO67, ILE68, TRP69, GLY91, VAL92, LEU93, CYS95, SER96, HIS97 | -5.22 |
| Jatrorrhizine |  | GLN26, GLY66, PRO67, ILE68, TRP69, GLY91, VAL92, LEU93, CYS95, SER96, HIS97 | -7.52 |
| Coptisine |  | GLN26, GLY66, PRO67, ILE68, TRP69, GLY91, VAL92, CYS95 | -5.57 |
| 8-oxyberberine |  | GLN26, PRO67, ILE68, TRP69, GLY91, VAL92, LEU93, CYS95, SER96, HIS97 | -5.35 |
| 1-Methoxyberberine |  | GLN26, GLY66, PRO67, ILE68, TRP69, GLY91, VAL92, CYS95, HIS97 | -4.93 |
| 13-Methylberberine |  | GLN26, GLY66, PRO67, ILE68, TRP69, GLY91, VAL92, LEU93, CYS95, HIS97 | -5.72 |
| 13-Methylpalmatine |  | GLN26, GLY66, PRO67, ILE68, TRP69,VAL92, CYS95, SER96, HIS97 | -5.66 |

Protein:SR-A1 SRCR domain (Msr1); Flexible residues: ILE68; Center grid box: X=16.009, Y=-18.603, Z=-3.57; Number of points: X=60, Y=Z=40.

_
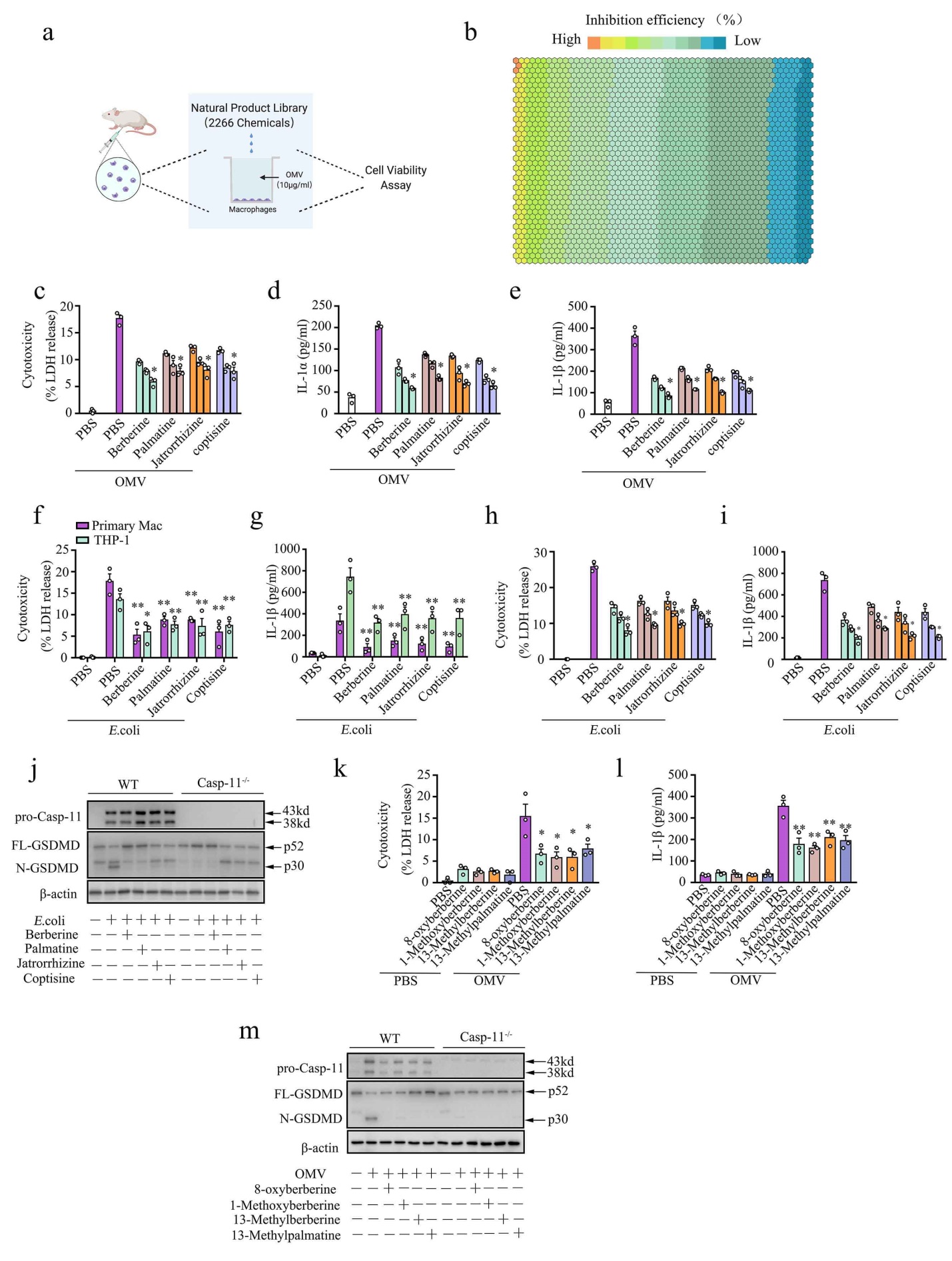
_

**Figure S1. Identification of berberine alkaloids as inhibitors of caspase-11 activation.** **a** and **b** Strategy of screening (**a**) and heatmap of cell viability changes (**b**) in mouse peritoneal macrophages stimulated with outer membrane vesicle (OMV, 10 μg/ml) in the presence of 2, 266 chemicals (5 μM) from a natural product library. **c-e** Cytotoxicity (**c**) as well as medium levels of IL-1α (**d**) and IL-1β (**e**) in macrophages treated with various doses of berberine alkaloids (0.5 μM, 1 μM, or 2 μM) and OMV (10μg/ml) (*versus* 0.5 μM + OMV groups). **f** and **g** % LDH (**f**) and IL-1β release (**g**) in macrophages and THP-1 cells treated with berberine alkaloids (5 μM) and challenged with *E.coli* (MOI 10) (*vs* PBS + *E.coli* groups). **h** and **i** %LDH (**h**) and IL-1β (**i**) in the medium of macrophages treated with various doses of berberine alkaloids (0.5 μM, 1 μM, or 2 μM) and challenged with *E.coli* (MOI 10) (*versus* 0.5 μM + *E.coli* groups). **j** Western blotting indicating caspase-11 and activation of GSDMD in macrophages (WT and *Casp11*^-/-^) treated with berberine alkaloids (2 μM) and challenged with *E.coli* (MOI 10). **k** and **l** Cytotoxicity (**k**) and medium levels of IL-1β (**l**) in macrophages treated with the derivatives of berberine alkaloids (2 μM) and challenged with OMV (10 μg/ml) (*versus* PBS + OMV groups). **m** Western blotting indicating caspase-11 and activation of GSDMD in macrophages (WT and *Casp11*^-/-^) treated with the derivatives of berberine alkaloids (2 μM) and OMV (10μg/ml). *p < 0.05; **p < 0.01. Data are shown as mean ± SEM.


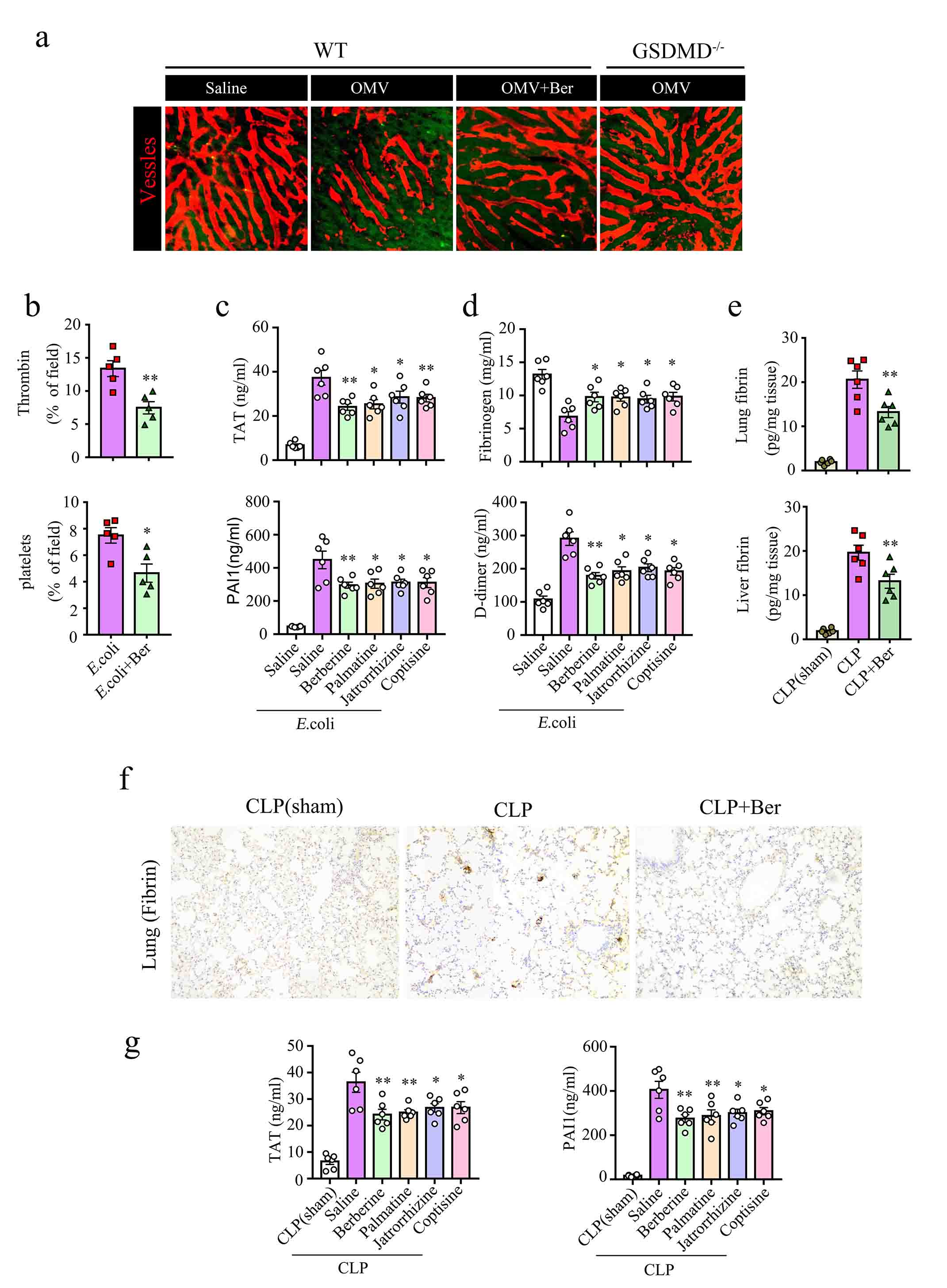


**Figure S2.** **Berberine alkaloids attenuate coagulation activation in bacterial sepsis. a** Representative SD-IVM images indicating vessel occlusion (red) in the liver microvasculature of LPS-primed mice treated with berberine (5 mg/kg) 30 mins prior to a challenge of OMV for 6 hours. **b-d**. Berberine (5 mg/kg) was intraperitoneally introduced to mice 30 mins prior to a challenge of *E.coli* for 4 hours (*versus* Saline + *E.coli* groups). **b** Quantitative analyses of thrombin-loaded and platelets-aggregated microvasculature in the liver using ImageJ. **c** and **d** Plasma levels of TAT complexes and PAI-1 (**c**) as well as fibrinogen and D-dimer (**d**) in mice treated with berberine alkaloids 30 mins before challenged with *E.coli* for 12 hours (*versus* Saline + *E.coli* groups). **e-g**. Mice were treated with berberine alkaloids 30 mins before a challenge of CLP for 12 hours (*versus* Saline + CLP groups). **e** Fibrin level of the liver and the lung. **f** Immunohistochemical staining of fibrin in the lung. **g** Levels of TAT and PAI-1 in the plasma of mice. *p < 0.05; **p < 0.01. Data are shown as mean ± SEM.


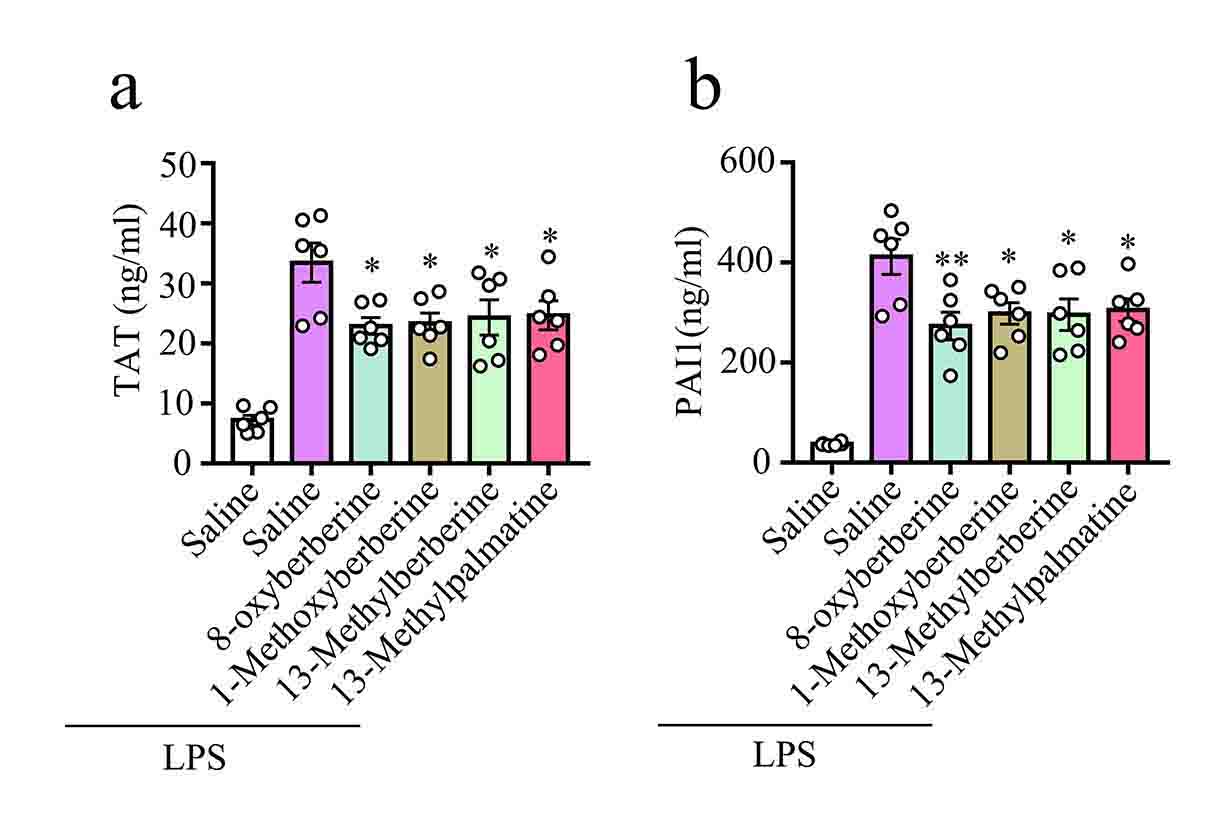


**Figure S3.** **The derivatives of berberine alkaloids attenuate endotoxin-mediated coagulation activation**. **a** and **b** Plasma TAT (**a**) and PAI-1 (**b**) in mice treated with the derivatives (5 mg/kg), such as 8-oxyberberine, 1-methoxyberberine, 13-methylberberine, and 13-methylpalmatine, 30 mins prior to a challenge of LPS (*versus* Saline + LPS group).


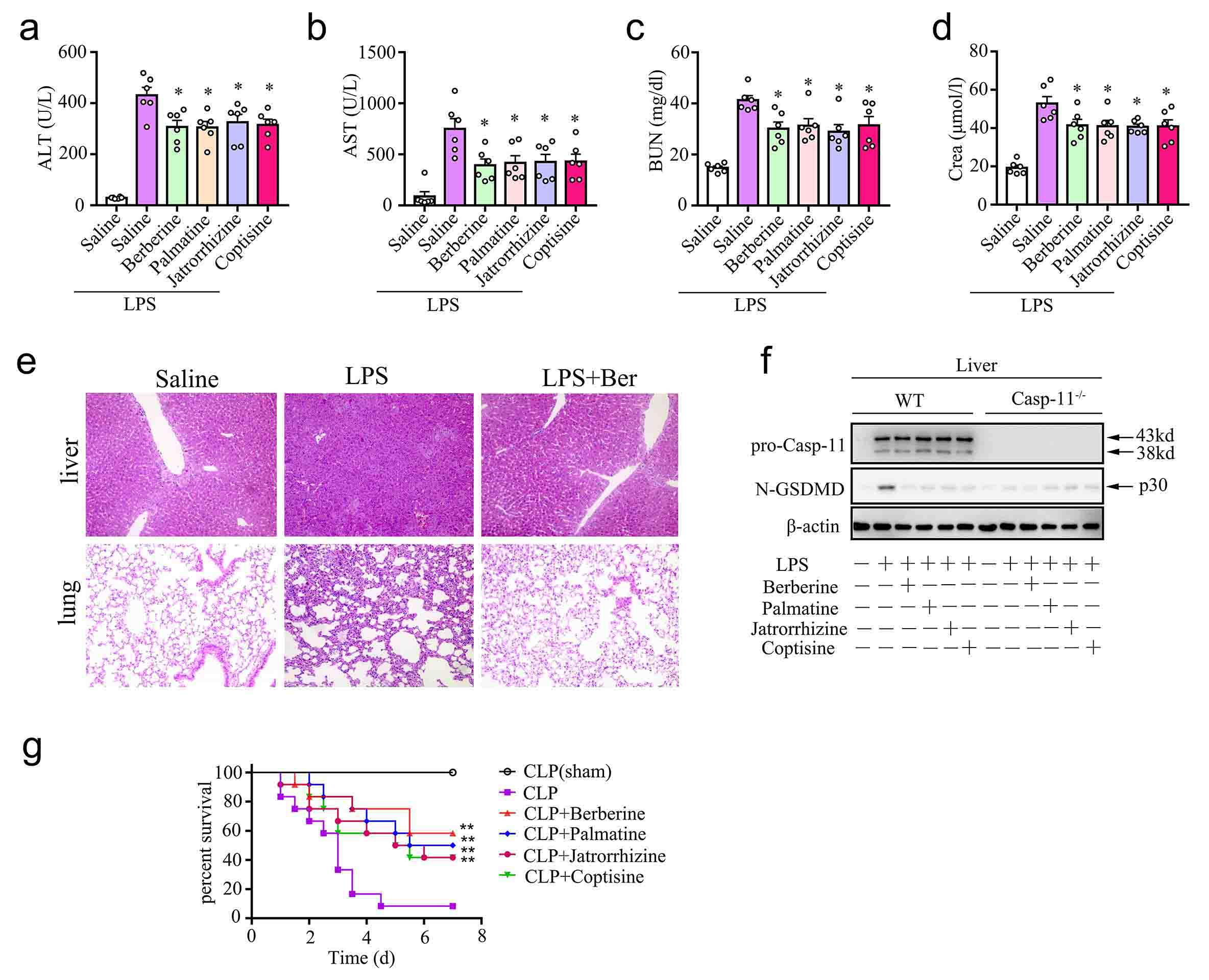


**Figure S4. Berberine alkaloids protect against organ dysfunction and death in sepsis by inhibiting caspase-11 pathway a-f**. Mice were primed with LPS (0.4 mg/kg) for 7 hours and subsequently administrated with berberine alkaloids (5 mg/kg) 30mins before a challenge of LPS (10 mg/kg) for 8 hours (*versus* Saline + LPS groups). **a** and **b** Plasma levels of alanine transaminase (ALT) and aspartate aminotransferase (AST). **c** and **d** Plasma levels of blood urea nitrogen (BUN) and creatinine (CREA). **e** H&E staining of the livers and lung of mice. **f** Western blotting indicating caspase-11 and activation of GSDMD in the livers of mice. **g** Kaplan-Meier survival plots in mice treated with berberine alkaloids (5 mg/kg) 30 mins prior to a challenge of CLP (*versus* Saline + CLP). *p < 0.05; **p < 0.01. Data are shown as mean ± SEM.


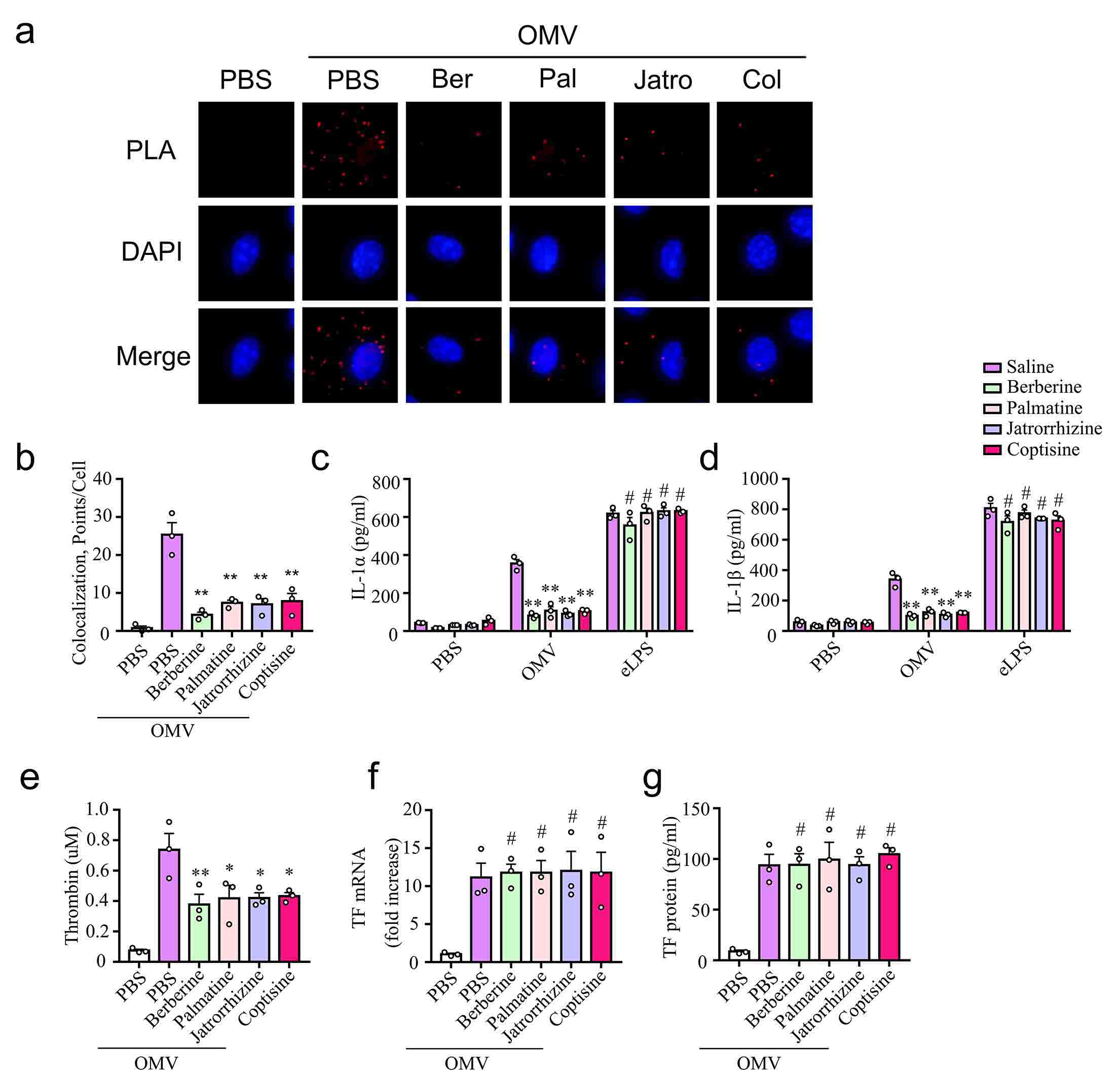


**Figure S5. Berberine alkaloids suppress endocytosis of LPS and inhibit TF activity. a and b** The binding of caspase-11 and LPS assessed by PLA assays in macrophages treated with berberine alkaloids (2 μM) and OMV (10 μg/ml) (*versus* Saline + OMV group). **c** and **d** Medium levels of IL-1α (**c**) and IL-1β (**d**) (*versus* Saline + OMV or LPS electrorotation groups, respectively). **e-g** Cells were treated with berberine alkaloids (2 μM) and OMV (10 μg/ml) (*versus* Saline + OMV group). **e** Thrombin formation. **f** and **g** TF mRNA and protein level. *p < 0.05; **p < 0.01, # no significant difference. Data are shown as mean ± SEM.


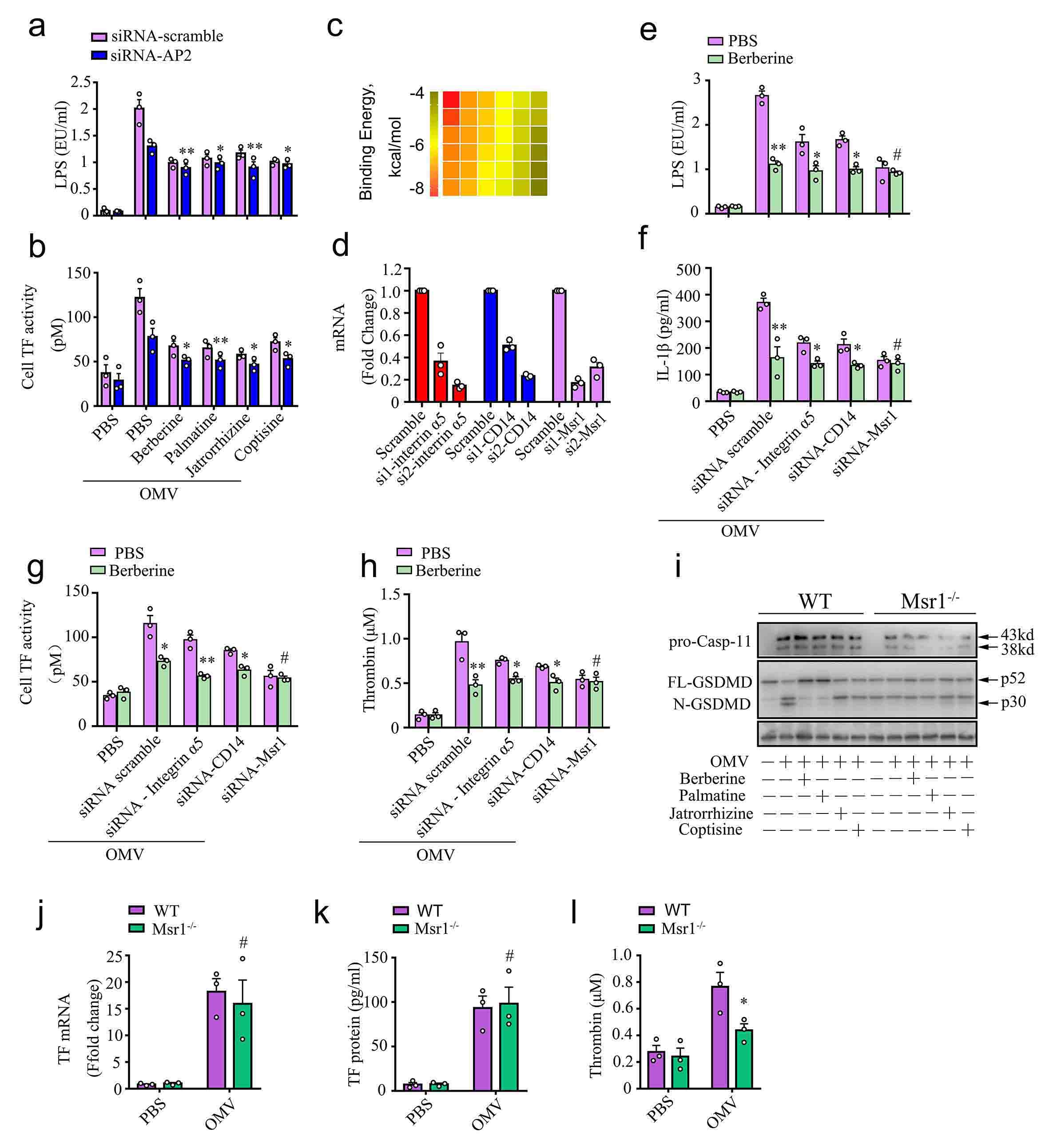


**Figure S6. Berberine alkaloids target Msr1 to inhibit endocytosis of LPS and restore caspase-11-mediated coagulation activation**

**a** and **b** Levels of cytosolic LPS (**a**) and TF activity (**b**) in macrophages (human TF+) treated with or without berberine (2 μM) and/or AP2 siRNA prior to a challenge of OMV (10 μg/ml) (*versus* PBS + OMV groups in AP2 siRNA-treated cells). **c** Heatmap of binding energy between berberine and proteins of endocytosis-related and LPS-associated factors. **d** Analysis of mRNA level of indicated genes in macrophages after treated with corresponding siRNA. **e**-**h**. Macrophages were treated with the scramble or siRNA targeting Integrin α5, CD14 or Msr1 in the presence or absence of berberine (2 μM) prior to a challenge of OMV (10 μg/ml) (PBS groups *versus* Berberine groups). **e** and **f** Levels of cytosolic LPS (**e**) and IL-1β release (**f**). **g** and **h** Levels of cell TF activity (**g**) and thrombin generation (**h**) in macrophages (expressing human TF). **i** Western blotting indicating caspase-11 and activation of GSDMD. **j** and **k** TF mRNA and protein levels. **l** Level of thrombin production in WT and *Msr1*-deficient macrophages treated with PBS or OMV (10 μg/ml). *p < 0.05; **p < 0.01, # no significant difference. Data are shown as mean ± SEM.
